# 5,8-Bis[bis­(pyridin-2-yl)amino]-1,3,4,6,7,9,9b-hepta­aza­phenalen-2(1*H*)-one dimethyl sulfoxide monosolvate dihydrate

**DOI:** 10.1107/S1600536814005698

**Published:** 2014-03-19

**Authors:** Anke Schwarzer, Edwin Kroke

**Affiliations:** a Institut für Anorganische Chemie, TU Bergakademie Freiberg, Leipziger Strasse 29, D-09596 Freiberg/Sachsen, Germany

## Abstract

In the asymmetric unit of the title compound, C_26_H_17_N_13_O·C_2_H_6_OS·2H_2_O, there is one independent hepta­zine-based main mol­ecule, one dimethyl sulfoxide mol­ecule and two water mol­ecules as solvents. The tri-*s*-triazine unit is substituted with two dipyridyl amine moieties and a carbonylic O atom. As indicated by the bond lengths in this acid unit of the hepta­zine derivative [C=O = 1.213 (2) Å, while the adjacent C—N(H) bond = 1.405 (2) Å] it is best described by the keto form. The cyameluric nucleus is close to planar (r.m.s. deviation = 0.061 Å) and the pyridine rings are inclined to its mean plane by dihedral angles varying from 47.47 (5) to 70.22 (5)°. The host and guest mol­ecules are connected *via* N—H⋯O, O—H⋯O and O—H⋯N hydrogen bonds, forming a four-membered inversion dimer-like arrangement enclosing an *R*_4_^4^(24) ring motif. These arrangements stack along [1-10] with a weak π–π inter­action [inter-centroid distance = 3.8721 (12) Å] involving adjacent pyridine rings. There are also C—H⋯N and C—H⋯O hydrogen bonds and C—H⋯π inter­actions present within the host mol­ecule and linking inversion-related mol­ecules, forming a three-dimensional structure.

## Related literature   

For a review of tri-*s*-triazines, see: Schwarzer *et al.* (2013 ▶). For crystal structures and a comprehensive analysis of cyameluric acid, see: Sattler & Schnick (2006 ▶); Wagler *et al.* (2006 ▶); Seyfarth *et al.* (2008 ▶). For the synthesis of unsymmetrically substituted tri-*s*-triazines, see: Schwarzer & Kroke (2010 ▶, 2011 ▶). For standard bond-length data, see: Allen *et al.* (1987 ▶). 
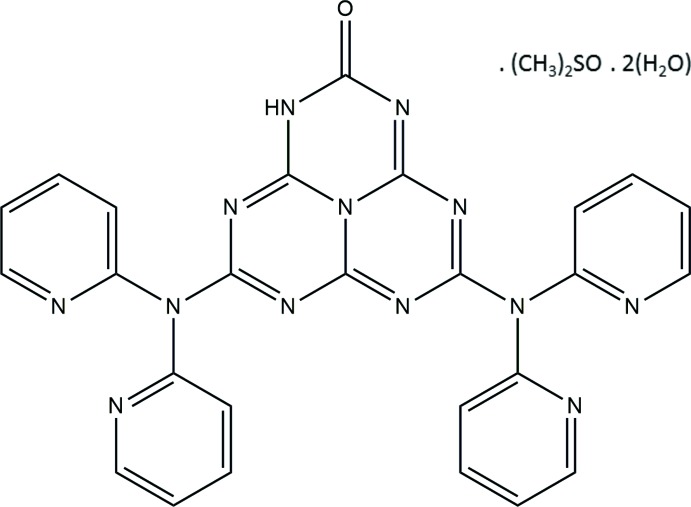


## Experimental   

### 

#### Crystal data  

C_26_H_17_N_13_O·C_2_H_6_OS·2H_2_O
*M* *_r_* = 641.69
Triclinic, 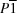

*a* = 10.6534 (2) Å
*b* = 11.6791 (2) Å
*c* = 12.5591 (2) Å
α = 68.488 (1)°
β = 86.537 (1)°
γ = 86.693 (1)°
*V* = 1450.06 (4) Å^3^
*Z* = 2
Mo *K*α radiation
μ = 0.17 mm^−1^
*T* = 100 K
0.26 × 0.25 × 0.21 mm

#### Data collection  

Bruker SMART CCD area-detector diffractometer
22692 measured reflections
5662 independent reflections
4333 reflections with *I* > 2σ(*I*)
*R*_int_ = 0.043

#### Refinement  

*R*[*F* ^2^ > 2σ(*F* ^2^)] = 0.039
*wR*(*F* ^2^) = 0.115
*S* = 0.97
5662 reflections
436 parameters
H atoms treated by a mixture of independent and constrained refinement
Δρ_max_ = 0.52 e Å^−3^
Δρ_min_ = −0.44 e Å^−3^

### 

Data collection: *SMART* (Bruker, 2007 ▶); cell refinement: *SAINT* (Bruker, 2007 ▶); data reduction: *SAINT*; program(s) used to solve structure: *SHELXS97* (Sheldrick, 2008 ▶); program(s) used to refine structure: *SHELXL97* (Sheldrick, 2008 ▶); molecular graphics: *SHELXTL* (Sheldrick, 2008 ▶); software used to prepare material for publication: *SHELXTL*.

## Supplementary Material

Crystal structure: contains datablock(s) global, I. DOI: 10.1107/S1600536814005698/su2711sup1.cif

Structure factors: contains datablock(s) I. DOI: 10.1107/S1600536814005698/su2711Isup2.hkl

Supporting information file. DOI: 10.1107/S1600536814005698/su2711Isup3.cml

CCDC reference: 991459

Additional supporting information:  crystallographic information; 3D view; checkCIF report

## Figures and Tables

**Table 1:** Hydrogen-bond geometry (Å, °) *Cg*1 and *Cg*2 are the centroids of the N12/C17–C21 and N13/C22–C26 rings, respectively.

*D*—H⋯*A*	*D*—H	H⋯*A*	*D*⋯*A*	*D*—H⋯*A*
N1—H1*N*⋯O3^i^	0.87 (2)	1.84 (2)	2.706 (2)	169 (2)
O3—H1*O*⋯O2	0.89 (4)	1.92 (3)	2.755 (2)	157 (3)
O3—H2*O*⋯N12^ii^	0.89 (3)	1.99 (3)	2.859 (2)	165 (3)
O4—H3*O*⋯N6^iii^	0.94 (4)	2.04 (4)	2.904 (3)	151 (3)
O4—H4*O*⋯N5^iii^	1.02 (3)	2.55 (4)	3.206 (3)	122 (2)
C9—H9⋯N10^iv^	0.95	2.55	3.313 (3)	137
C21—H21⋯O2^v^	0.95	2.41	3.291 (3)	154
C23—H23⋯O1^vi^	0.95	2.55	3.426 (2)	153
C27—H27*B*⋯*Cg*2	0.98	2.76	3.647 (3)	150
C28—H28*A*⋯*Cg*1^vii^	0.98	2.73	3.463 (2)	132

Symmetry codes: (i) 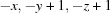; (ii) 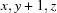; (iii) 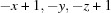; (iv) 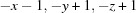; (v) 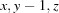; (vi) 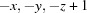; (vii) 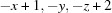.

**Fig. 1.**
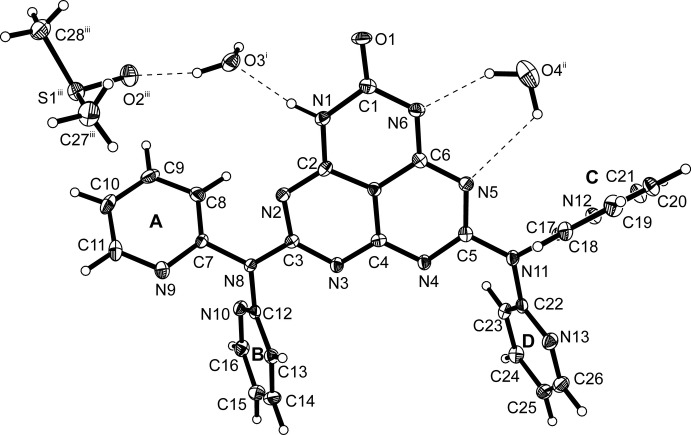
A view of the molecular structure of the title compound, with atom labelling. The displacement ellipsoids are drawn at the 50% probability level. The hydrogen bonds are shown as dashed lines (see Table 1 for details).

**Fig. 2.**
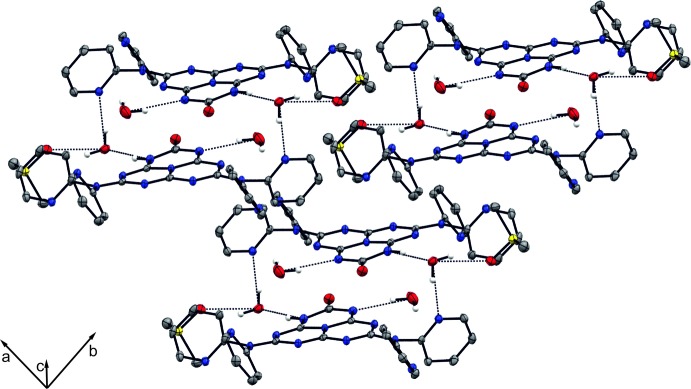
A partial view along the c axis of the crystal packing of the title compound. Hydrogen bonds are shown as dashed lines (see Table 1 for details; H atoms not involved in hydrogen bonding have been omitted for clarity).

## supplementary crystallographic information

### 1. Comment

Cyameluric acid is described to crystallize with water (Sattler & Schnick, 2006), dimethylsulfoxide (Wagler *et al.*, 2006) and free of solvent (Seyfarth *et al.*, 2008). All structures reveal the keto form of the cyameluric nucleus independent of the co-crystallizing solvent. Molecular derivatives are rarely described especially unsymmetrically substituted ones. Herein, we describe the crystal structure of an unsymmetrically substituted cyameluric acid derivative.

The molecular structure of the host and guest molecules of the title compound are illustrated in Fig. 1. The bond lengths (Allen *et al.*, 1987) and angles are in the range of expected values. As indicated by the C—N bond lengths of the heptazine core the keto form is preferred rather than the hydroxyl form. The C1-O1 bond length [1.213 (2) Å] is a typical C═O bond while the adjacent C1-N1 bond length [1.405 (2) Å] represents a typical C—N single bond. Besides, the bond length of C1—N6 [1.371 (2) Å] is close to a single C—N bond but still indicates the conjugation as expected for the C_6_N_7_ core. Additionally, the C—N bond lengths of the inner heptazine core (N7—C2/C4/C6) are significantly shorter on the protonated site of the molecule [1.375 (2) Å in contrast to 1.401 (2) and 1.410 (2) Å]. Furthermore, the N1 hydrogen atom was clearly visible in a difference electron-density map.

Neither the unsymmetrical substitution of the C_6_N_7_ core nor the keto form and the adjacent C—N single bond character influence the planarity of the host molecule. The fit of the 13-membered ring system to a plane leads to a r.m.s. deviation of 0.061 Å indicating nearly perfect planarity. The pyridyl moieties reveal a twisting relating to the heptazine ring [47.47 (5) - 70.22 (5)°; average: 60.72°] and are nearly perpendicular to one other (average pyridine-pyridine dihedral angle: 82.02°) except for rings N10/C12-C16 and N13/C22-C26 which are inclined to one another by 12.57 (8)°.

In the crystal, the host and guest molecules are linked *via* hydrogen bonds. The hydrogen atom H1N located at N1 interacts with one water molecule (d = 1.84 Å, θ = 170°). This water molecule is coordinated to the dimethyl sulfoxide [O···O: 2.755 (2) Å] and the pyridine ring of an adjacent host molecule [O···N: 2.859 (2) Å]. Additionally to the O–H···O interaction, the DMSO molecule shows C–H···O contacts with donor-acceptor distances of about 2.5 Å and C—H···π interactions with adjacent pyridyl units. The hydrogen-centroid distance is in the range of 2.7 Å. Also, the O–H···S reveals a weak hydrogen bond [O···S: 3.9505 (17) Å]. The second water molecule is located close to the C_6_N_7_ core with O···N distance of 2.904 (2) Å and 3.206 (2) Å. This water molecule also reveals a possible intermolecular O—H···π contact with a hydrogen-centroid distance of 2.91 Å.

The crystal packing does not represent a layered structure as it is known for other heptazine derivatives (Schwarzer *et al.*, 2013). This is indicated by the distances between adjacent C_6_N_7_-cores and the great offset to one another. A weak π···π interaction occurs between adjacent pyridyl units. The centroid *Cg*5 of the ring N10/C12—C16 reveals a distance of 3.8721 (12) Å to the centroid *Cg*7 of the ring N13/C22–26 (symmetry code: -*x*, -*y*, -*z*+2).

To sum up, the title cyameluric compound occurs in its keto form as it is known from other derivatives. In the crystal the interactions of the host–guest compound include O–H···O/N, N–H···O, C–H···O/N/π/ and π···π stacking.

### 2. Experimental

α,α'-Dipyridylamine (0.12 g, 0.7 mmol) in 20 ml THF was added to cyameluric chloride (0.1 g, 0.36 mmol) dessolved in 15 ml THF. The mixture was refluxed for 8 h and stirred overnight at room temperature to give a yellow solution and a pale white precipitate. The solid (α,α'-dipyridylamine hydrochloride) was separated *via* suction filtration. Adding aqueous THF leads to a crystalline solid which was seperated *via* filtration and dried under air. Colourless prismatic crystals suitable for X-ray diffraction analysis were taken from that batch. Spectroscopic data for the title compound are available in the archived CIF.

### 3. Refinement

The NH and OH H atoms were located in a difference Fourier map and freely refined. The C-bound H atoms were positioned geometrically and allowed to ride on their parent atoms: C–H = 0.95 and 0.98 Å for aryl and aliphatic H atom, respectively, with *U*_iso_(H) =1.2*U*_eq_(C).

### Crystal data

**Table:**

C_26_H_17_N_13_O·C_2_H_6_OS·2H_2_O	*Z* = 2
*M**_r_* = 641.69	*F*(000) = 668
Triclinic, *P*1	*D*_x_ = 1.470 Mg m^−^^3^
Hall symbol: -P 1	Mo *K*α radiation, λ = 0.71073 Å
*a* = 10.6534 (2) Å	Cell parameters from 852 reflections
*b* = 11.6791 (2) Å	θ = 2.5–25.2°
*c* = 12.5591 (2) Å	µ = 0.17 mm^−^^1^
α = 68.488 (1)°	*T* = 100 K
β = 86.537 (1)°	Prism, colourless
γ = 86.693 (1)°	0.26 × 0.25 × 0.21 mm
*V* = 1450.06 (4) Å^3^	

### Data collection

**Table:**

Bruker SMART CCD area-detector diffractometer	4333 reflections with *I* > 2σ(*I*)
Radiation source: fine-focus sealed tube	*R*_int_ = 0.043
Graphite monochromator	θ_max_ = 26.0°, θ_min_ = 2.5°
phi and ω scans	*h* = −13→13
22692 measured reflections	*k* = −14→14
5662 independent reflections	*l* = −15→15

### Refinement

**Table:**

Refinement on *F*^2^	Primary atom site location: structure-invariant direct methods
Least-squares matrix: full	Secondary atom site location: difference Fourier map
*R*[*F*^2^ > 2σ(*F*^2^)] = 0.039	Hydrogen site location: inferred from neighbouring sites
*wR*(*F*^2^) = 0.115	H atoms treated by a mixture of independent and constrained refinement
*S* = 0.97	*w* = 1/[σ^2^(*F*_o_^2^) + (0.0704*P*)^2^ + 0.3491*P*] where *P* = (*F*_o_^2^ + 2*F*_c_^2^)/3
5662 reflections	(Δ/σ)_max_ = 0.003
436 parameters	Δρ_max_ = 0.52 e Å^−^^3^
0 restraints	Δρ_min_ = −0.44 e Å^−^^3^

### Special details

**Table:**

Experimental. Spectroscopic data for the title compound: IR (KBr): *ν*_max_ (cm^-1^) 3379 (w, NH), 3053 (w, C_Ar_H), 1654, 1649, 1643, 1634 (s, C=O, C=C, C=N), 1590 (s, py), 1408, 817 (s, heptazine ring), 744, 724 (δ_CHoop_, pyridyl rings).
Geometry. Bond distances, angles etc. have been calculated using the rounded fractional coordinates. All su's are estimated from the variances of the (full) variance-covariance matrix. The cell esds are taken into account in the estimation of distances, angles and torsion angles
Refinement. Refinement of *F*^2^ against ALL reflections. The weighted *R*-factor *wR* and goodness of fit *S* are based on *F*^2^, conventional *R*-factors *R* are based on *F*, with *F* set to zero for negative *F*^2^. The threshold expression of *F*^2^ > σ(*F*^2^) is used only for calculating *R*-factors(gt) *etc*. and is not relevant to the choice of reflections for refinement. *R*-factors based on *F*^2^ are statistically about twice as large as those based on *F*, and *R*- factors based on ALL data will be even larger.

### Fractional atomic coordinates and isotropic or equivalent isotropic displacement parameters (Å^2^)

**Table:**

	*x*	*y*	*z*	*U*_iso_*/*U*_eq_	
O1	0.05786 (13)	0.15490 (13)	0.22548 (11)	0.0218 (4)	
N1	−0.00053 (15)	0.25898 (15)	0.34166 (14)	0.0158 (5)	
N2	−0.07731 (14)	0.35788 (14)	0.46186 (13)	0.0138 (5)	
N3	−0.03158 (14)	0.26735 (14)	0.66201 (13)	0.0143 (5)	
N4	0.10816 (14)	0.10167 (14)	0.72397 (13)	0.0143 (5)	
N5	0.19000 (14)	0.00471 (14)	0.59246 (13)	0.0146 (5)	
N6	0.12594 (15)	0.07445 (15)	0.40763 (13)	0.0156 (5)	
N7	0.04826 (14)	0.17601 (14)	0.53204 (13)	0.0128 (5)	
N8	−0.16964 (14)	0.43367 (14)	0.59394 (13)	0.0135 (5)	
N9	−0.25278 (15)	0.63147 (14)	0.50174 (14)	0.0173 (5)	
N10	−0.31413 (15)	0.41777 (15)	0.74385 (14)	0.0169 (5)	
N11	0.26055 (14)	−0.05485 (14)	0.77349 (13)	0.0145 (5)	
N12	0.34523 (15)	−0.24585 (14)	0.77874 (14)	0.0171 (5)	
N13	0.35974 (15)	−0.06482 (15)	0.93686 (14)	0.0182 (5)	
C1	0.06245 (17)	0.16025 (17)	0.31984 (16)	0.0156 (6)	
C2	−0.01201 (17)	0.26748 (17)	0.44541 (15)	0.0131 (5)	
C3	−0.09001 (16)	0.34885 (16)	0.57275 (15)	0.0124 (5)	
C4	0.04141 (17)	0.18114 (17)	0.64196 (15)	0.0125 (5)	
C5	0.18183 (17)	0.01959 (17)	0.69395 (16)	0.0136 (5)	
C6	0.12277 (17)	0.08334 (16)	0.50897 (16)	0.0133 (5)	
C7	−0.25117 (17)	0.51484 (17)	0.50902 (15)	0.0130 (5)	
C8	−0.32791 (18)	0.47066 (19)	0.44910 (17)	0.0195 (6)	
C9	−0.41163 (19)	0.5526 (2)	0.37663 (18)	0.0236 (7)	
C10	−0.41555 (19)	0.6745 (2)	0.36704 (17)	0.0233 (6)	
C11	−0.33584 (19)	0.70857 (18)	0.43081 (17)	0.0215 (6)	
C12	−0.19442 (17)	0.43318 (16)	0.70828 (16)	0.0136 (5)	
C13	−0.10072 (18)	0.45271 (17)	0.77047 (16)	0.0156 (5)	
C14	−0.13402 (19)	0.45589 (19)	0.87735 (17)	0.0209 (6)	
C15	−0.25851 (19)	0.44010 (19)	0.91663 (17)	0.0205 (6)	
C16	−0.34422 (19)	0.42104 (18)	0.84769 (17)	0.0190 (6)	
C17	0.35980 (17)	−0.12526 (17)	0.73935 (15)	0.0137 (5)	
C18	0.46274 (18)	−0.06784 (18)	0.67347 (17)	0.0177 (6)	
C19	0.55494 (19)	−0.14012 (19)	0.64387 (18)	0.0220 (6)	
C20	0.54238 (19)	−0.26591 (19)	0.68373 (18)	0.0221 (6)	
C21	0.43729 (19)	−0.31484 (18)	0.75083 (18)	0.0212 (6)	
C22	0.25133 (18)	−0.07091 (16)	0.89261 (16)	0.0141 (5)	
C23	0.13735 (18)	−0.09643 (17)	0.95411 (16)	0.0158 (5)	
C24	0.13640 (18)	−0.11568 (18)	1.06925 (17)	0.0180 (6)	
C25	0.24749 (19)	−0.11014 (17)	1.11841 (17)	0.0184 (6)	
C26	0.35607 (19)	−0.08504 (18)	1.04889 (17)	0.0200 (6)	
S1	0.32151 (5)	0.27219 (5)	0.92748 (4)	0.0209 (2)	
O2	0.32255 (14)	0.41053 (13)	0.88182 (13)	0.0255 (4)	
C27	0.1615 (2)	0.2350 (2)	0.9319 (2)	0.0311 (7)	
C28	0.3406 (2)	0.2251 (2)	1.07765 (18)	0.0294 (7)	
O3	0.13761 (14)	0.59490 (15)	0.83566 (12)	0.0225 (5)	
O4	0.71369 (19)	0.14203 (16)	0.57023 (18)	0.0421 (6)	
H1N	−0.041 (2)	0.314 (2)	0.286 (2)	0.023 (6)*	
H8	−0.32310	0.38630	0.45760	0.0230*	
H9	−0.46580	0.52570	0.33390	0.0280*	
H10	−0.47200	0.73300	0.31750	0.0280*	
H11	−0.33960	0.79230	0.42440	0.0260*	
H13	−0.01630	0.46360	0.74070	0.0190*	
H14	−0.07260	0.46870	0.92320	0.0250*	
H15	−0.28420	0.44240	0.98960	0.0250*	
H16	−0.42930	0.40960	0.87530	0.0230*	
H18	0.46990	0.01880	0.64920	0.0210*	
H19	0.62600	−0.10370	0.59670	0.0260*	
H20	0.60490	−0.31780	0.66530	0.0260*	
H21	0.42940	−0.40160	0.77880	0.0250*	
H23	0.06260	−0.10050	0.91830	0.0190*	
H24	0.06010	−0.13260	1.11440	0.0220*	
H25	0.24920	−0.12320	1.19760	0.0220*	
H26	0.43250	−0.08200	1.08280	0.0240*	
H27A	0.11060	0.27510	0.97690	0.0470*	
H27B	0.15400	0.14550	0.96750	0.0470*	
H27C	0.13160	0.26390	0.85380	0.0470*	
H28A	0.42560	0.24300	1.09130	0.0440*	
H28B	0.32820	0.13640	1.11420	0.0440*	
H28C	0.27850	0.26990	1.11000	0.0440*	
H1O	0.180 (3)	0.524 (3)	0.847 (3)	0.061 (10)*	
H2O	0.195 (3)	0.650 (3)	0.827 (2)	0.048 (8)*	
H3O	0.767 (3)	0.072 (3)	0.603 (3)	0.059 (9)*	
H4O	0.693 (3)	0.141 (3)	0.492 (3)	0.0700*	

### Atomic displacement parameters (Å^2^)

**Table:**

	*U*^11^	*U*^22^	*U*^33^	*U*^12^	*U*^13^	*U*^23^
O1	0.0260 (8)	0.0294 (8)	0.0141 (7)	0.0009 (6)	−0.0024 (6)	−0.0130 (6)
N1	0.0175 (9)	0.0177 (8)	0.0118 (8)	0.0017 (7)	−0.0016 (7)	−0.0051 (7)
N2	0.0132 (8)	0.0155 (8)	0.0122 (8)	0.0006 (6)	−0.0010 (6)	−0.0045 (7)
N3	0.0135 (8)	0.0145 (8)	0.0135 (8)	0.0018 (6)	−0.0001 (6)	−0.0039 (7)
N4	0.0144 (8)	0.0143 (8)	0.0129 (8)	0.0025 (6)	−0.0001 (6)	−0.0041 (7)
N5	0.0143 (8)	0.0163 (8)	0.0136 (8)	0.0010 (6)	−0.0004 (6)	−0.0061 (7)
N6	0.0150 (8)	0.0192 (8)	0.0144 (8)	−0.0005 (7)	−0.0002 (6)	−0.0082 (7)
N7	0.0121 (8)	0.0132 (8)	0.0131 (8)	0.0003 (6)	0.0000 (6)	−0.0049 (6)
N8	0.0140 (8)	0.0143 (8)	0.0125 (8)	0.0030 (6)	−0.0034 (6)	−0.0052 (7)
N9	0.0156 (8)	0.0158 (8)	0.0186 (9)	0.0004 (6)	0.0015 (7)	−0.0047 (7)
N10	0.0152 (8)	0.0188 (8)	0.0158 (8)	0.0021 (7)	−0.0017 (7)	−0.0056 (7)
N11	0.0135 (8)	0.0155 (8)	0.0130 (8)	0.0038 (6)	−0.0002 (6)	−0.0042 (7)
N12	0.0167 (8)	0.0166 (8)	0.0188 (9)	0.0002 (7)	0.0007 (7)	−0.0076 (7)
N13	0.0144 (8)	0.0222 (9)	0.0176 (9)	0.0015 (7)	−0.0013 (7)	−0.0070 (7)
C1	0.0120 (9)	0.0193 (10)	0.0173 (10)	−0.0019 (7)	0.0014 (8)	−0.0089 (8)
C2	0.0096 (9)	0.0153 (9)	0.0125 (9)	−0.0021 (7)	−0.0009 (7)	−0.0027 (8)
C3	0.0099 (9)	0.0142 (9)	0.0129 (9)	−0.0019 (7)	0.0000 (7)	−0.0045 (8)
C4	0.0105 (9)	0.0160 (9)	0.0113 (9)	−0.0030 (7)	0.0019 (7)	−0.0054 (8)
C5	0.0110 (9)	0.0139 (9)	0.0148 (9)	−0.0016 (7)	0.0016 (7)	−0.0041 (8)
C6	0.0105 (9)	0.0132 (9)	0.0162 (10)	−0.0028 (7)	0.0020 (7)	−0.0054 (8)
C7	0.0120 (9)	0.0154 (9)	0.0100 (9)	0.0029 (7)	−0.0004 (7)	−0.0032 (8)
C8	0.0186 (10)	0.0212 (10)	0.0222 (11)	0.0034 (8)	−0.0032 (8)	−0.0123 (9)
C9	0.0192 (11)	0.0367 (13)	0.0188 (11)	0.0076 (9)	−0.0075 (8)	−0.0151 (10)
C10	0.0184 (11)	0.0302 (12)	0.0157 (10)	0.0094 (9)	−0.0029 (8)	−0.0028 (9)
C11	0.0213 (11)	0.0158 (10)	0.0216 (11)	0.0044 (8)	0.0016 (8)	−0.0012 (8)
C12	0.0167 (10)	0.0109 (9)	0.0118 (9)	0.0023 (7)	−0.0017 (7)	−0.0028 (7)
C13	0.0147 (9)	0.0153 (9)	0.0167 (10)	0.0000 (7)	−0.0006 (8)	−0.0060 (8)
C14	0.0212 (11)	0.0245 (11)	0.0203 (11)	−0.0003 (8)	−0.0053 (8)	−0.0116 (9)
C15	0.0238 (11)	0.0249 (11)	0.0138 (10)	0.0031 (8)	0.0016 (8)	−0.0091 (9)
C16	0.0155 (10)	0.0210 (10)	0.0189 (10)	0.0020 (8)	0.0002 (8)	−0.0060 (8)
C17	0.0130 (9)	0.0180 (9)	0.0110 (9)	0.0037 (7)	−0.0024 (7)	−0.0067 (8)
C18	0.0178 (10)	0.0145 (9)	0.0184 (10)	0.0013 (8)	−0.0003 (8)	−0.0036 (8)
C19	0.0155 (10)	0.0264 (11)	0.0217 (11)	−0.0003 (8)	0.0053 (8)	−0.0068 (9)
C20	0.0204 (11)	0.0247 (11)	0.0232 (11)	0.0060 (9)	0.0008 (9)	−0.0124 (9)
C21	0.0226 (11)	0.0156 (10)	0.0262 (11)	0.0022 (8)	0.0004 (9)	−0.0092 (9)
C22	0.0158 (10)	0.0114 (9)	0.0139 (9)	0.0029 (7)	−0.0016 (7)	−0.0036 (8)
C23	0.0135 (9)	0.0152 (9)	0.0161 (10)	0.0006 (7)	−0.0025 (8)	−0.0027 (8)
C24	0.0163 (10)	0.0180 (10)	0.0164 (10)	−0.0010 (8)	0.0029 (8)	−0.0028 (8)
C25	0.0247 (11)	0.0168 (10)	0.0126 (9)	0.0009 (8)	−0.0023 (8)	−0.0040 (8)
C26	0.0177 (10)	0.0246 (11)	0.0184 (10)	0.0018 (8)	−0.0043 (8)	−0.0085 (9)
S1	0.0203 (3)	0.0196 (3)	0.0227 (3)	0.0011 (2)	−0.0009 (2)	−0.0080 (2)
O2	0.0246 (8)	0.0193 (7)	0.0296 (8)	−0.0029 (6)	0.0004 (6)	−0.0054 (7)
C27	0.0256 (12)	0.0258 (12)	0.0402 (14)	−0.0063 (9)	−0.0084 (10)	−0.0083 (10)
C28	0.0347 (13)	0.0281 (12)	0.0238 (12)	−0.0006 (10)	−0.0074 (10)	−0.0068 (10)
O3	0.0202 (8)	0.0246 (8)	0.0217 (8)	−0.0014 (7)	−0.0065 (6)	−0.0065 (7)
O4	0.0475 (11)	0.0263 (9)	0.0543 (12)	0.0029 (8)	0.0038 (9)	−0.0183 (9)

### Geometric parameters (Å, º)

**Table:**

S1—O2	1.5037 (17)	C7—C8	1.382 (3)
S1—C27	1.776 (2)	C8—C9	1.379 (3)
S1—C28	1.781 (2)	C9—C10	1.382 (3)
O1—C1	1.213 (2)	C10—C11	1.370 (3)
O3—H2O	0.89 (3)	C12—C13	1.382 (3)
O3—H1O	0.89 (4)	C13—C14	1.381 (3)
O4—H4O	1.02 (3)	C14—C15	1.385 (3)
O4—H3O	0.94 (4)	C15—C16	1.376 (3)
N1—C1	1.405 (3)	C17—C18	1.380 (3)
N1—C2	1.341 (2)	C18—C19	1.381 (3)
N2—C2	1.304 (3)	C19—C20	1.379 (3)
N2—C3	1.357 (2)	C20—C21	1.377 (3)
N3—C3	1.336 (2)	C22—C23	1.386 (3)
N3—C4	1.324 (3)	C23—C24	1.379 (3)
N4—C4	1.319 (2)	C24—C25	1.383 (3)
N4—C5	1.349 (3)	C25—C26	1.384 (3)
N5—C6	1.328 (2)	C8—H8	0.9500
N5—C5	1.345 (2)	C9—H9	0.9500
N6—C1	1.371 (2)	C10—H10	0.9500
N6—C6	1.313 (2)	C11—H11	0.9500
N7—C6	1.410 (3)	C13—H13	0.9500
N7—C2	1.375 (2)	C14—H14	0.9500
N7—C4	1.401 (2)	C15—H15	0.9500
N8—C3	1.357 (3)	C16—H16	0.9500
N8—C12	1.442 (2)	C18—H18	0.9500
N8—C7	1.437 (2)	C19—H19	0.9500
N9—C11	1.343 (3)	C20—H20	0.9500
N9—C7	1.331 (3)	C21—H21	0.9500
N10—C16	1.338 (3)	C23—H23	0.9500
N10—C12	1.326 (2)	C24—H24	0.9500
N11—C5	1.355 (2)	C25—H25	0.9500
N11—C17	1.443 (3)	C26—H26	0.9500
N11—C22	1.436 (2)	C27—H27A	0.9800
N12—C21	1.345 (3)	C27—H27B	0.9800
N12—C17	1.326 (3)	C27—H27C	0.9800
N13—C26	1.337 (3)	C28—H28B	0.9800
N13—C22	1.328 (3)	C28—H28C	0.9800
N1—H1N	0.87 (2)	C28—H28A	0.9800
			
C27—S1—C28	98.35 (11)	N11—C17—N12	115.10 (16)
O2—S1—C27	105.83 (10)	N11—C17—C18	120.63 (18)
O2—S1—C28	105.64 (10)	N12—C17—C18	124.24 (19)
H1O—O3—H2O	106 (3)	C17—C18—C19	118.0 (2)
H3O—O4—H4O	105 (3)	C18—C19—C20	119.03 (19)
C1—N1—C2	123.79 (17)	C19—C20—C21	118.7 (2)
C2—N2—C3	114.65 (16)	N12—C21—C20	123.2 (2)
C3—N3—C4	116.84 (16)	N11—C22—N13	114.59 (16)
C4—N4—C5	115.90 (16)	N13—C22—C23	124.67 (18)
C5—N5—C6	117.09 (17)	N11—C22—C23	120.67 (17)
C1—N6—C6	120.25 (18)	C22—C23—C24	117.56 (18)
C2—N7—C6	120.51 (16)	C23—C24—C25	119.44 (18)
C4—N7—C6	120.49 (16)	C24—C25—C26	118.03 (18)
C2—N7—C4	118.86 (17)	N13—C26—C25	123.90 (19)
C7—N8—C12	115.08 (15)	C7—C8—H8	121.00
C3—N8—C12	121.72 (15)	C9—C8—H8	121.00
C3—N8—C7	122.29 (15)	C10—C9—H9	120.00
C7—N9—C11	116.01 (17)	C8—C9—H9	120.00
C12—N10—C16	116.68 (17)	C11—C10—H10	121.00
C5—N11—C17	119.34 (15)	C9—C10—H10	121.00
C17—N11—C22	116.41 (15)	C10—C11—H11	118.00
C5—N11—C22	124.25 (16)	N9—C11—H11	118.00
C17—N12—C21	116.81 (17)	C12—C13—H13	121.00
C22—N13—C26	116.40 (17)	C14—C13—H13	121.00
C1—N1—H1N	118.0 (16)	C13—C14—H14	121.00
C2—N1—H1N	118.0 (16)	C15—C14—H14	121.00
O1—C1—N1	118.93 (18)	C16—C15—H15	121.00
N1—C1—N6	117.56 (17)	C14—C15—H15	121.00
O1—C1—N6	123.51 (19)	N10—C16—H16	118.00
N1—C2—N2	121.13 (17)	C15—C16—H16	118.00
N2—C2—N7	122.59 (17)	C19—C18—H18	121.00
N1—C2—N7	116.28 (18)	C17—C18—H18	121.00
N2—C3—N8	115.78 (16)	C20—C19—H19	121.00
N2—C3—N3	127.18 (17)	C18—C19—H19	120.00
N3—C3—N8	117.03 (16)	C19—C20—H20	121.00
N3—C4—N7	119.27 (16)	C21—C20—H20	121.00
N3—C4—N4	120.57 (17)	C20—C21—H21	118.00
N4—C4—N7	120.16 (18)	N12—C21—H21	118.00
N5—C5—N11	115.28 (17)	C24—C23—H23	121.00
N4—C5—N5	127.81 (17)	C22—C23—H23	121.00
N4—C5—N11	116.88 (17)	C23—C24—H24	120.00
N5—C6—N6	120.51 (18)	C25—C24—H24	120.00
N6—C6—N7	121.08 (17)	C24—C25—H25	121.00
N5—C6—N7	118.40 (17)	C26—C25—H25	121.00
N8—C7—N9	113.94 (16)	N13—C26—H26	118.00
N8—C7—C8	121.48 (19)	C25—C26—H26	118.00
N9—C7—C8	124.35 (18)	S1—C27—H27B	109.00
C7—C8—C9	118.0 (2)	S1—C27—H27A	109.00
C8—C9—C10	119.0 (2)	H27A—C27—H27C	109.00
C9—C10—C11	118.2 (2)	S1—C27—H27C	109.00
N9—C11—C10	124.3 (2)	H27A—C27—H27B	109.00
N8—C12—N10	113.72 (16)	H27B—C27—H27C	110.00
N10—C12—C13	124.58 (18)	S1—C28—H28B	109.00
N8—C12—C13	121.64 (16)	S1—C28—H28C	109.00
C12—C13—C14	117.71 (18)	H28A—C28—H28C	110.00
C13—C14—C15	118.91 (19)	H28B—C28—H28C	109.00
C14—C15—C16	118.59 (19)	H28A—C28—H28B	109.00
N10—C16—C15	123.52 (19)	S1—C28—H28A	109.00
			
C2—N1—C1—O1	173.18 (18)	C7—N8—C12—N10	50.3 (2)
C2—N1—C1—N6	−6.7 (3)	C7—N8—C12—C13	−127.0 (2)
C1—N1—C2—N2	−176.25 (18)	C11—N9—C7—N8	−174.33 (16)
C1—N1—C2—N7	3.6 (3)	C11—N9—C7—C8	0.1 (3)
C3—N2—C2—N1	174.44 (17)	C7—N9—C11—C10	−0.5 (3)
C3—N2—C2—N7	−5.4 (3)	C16—N10—C12—N8	−177.59 (18)
C2—N2—C3—N3	8.6 (3)	C16—N10—C12—C13	−0.4 (3)
C2—N2—C3—N8	−172.50 (16)	C12—N10—C16—C15	0.5 (3)
C4—N3—C3—N2	−4.2 (3)	C17—N11—C5—N4	−166.75 (17)
C4—N3—C3—N8	176.88 (17)	C17—N11—C5—N5	11.2 (3)
C3—N3—C4—N4	175.98 (17)	C22—N11—C5—N4	14.3 (3)
C3—N3—C4—N7	−3.3 (3)	C22—N11—C5—N5	−167.75 (17)
C5—N4—C4—N3	−178.38 (17)	C5—N11—C17—N12	−110.5 (2)
C5—N4—C4—N7	0.9 (3)	C5—N11—C17—C18	71.5 (2)
C4—N4—C5—N5	−4.1 (3)	C22—N11—C17—N12	68.6 (2)
C4—N4—C5—N11	173.60 (17)	C22—N11—C17—C18	−109.5 (2)
C6—N5—C5—N4	3.6 (3)	C5—N11—C22—N13	−133.6 (2)
C6—N5—C5—N11	−174.08 (17)	C5—N11—C22—C23	49.4 (3)
C5—N5—C6—N6	−179.95 (17)	C17—N11—C22—N13	47.4 (2)
C5—N5—C6—N7	0.0 (3)	C17—N11—C22—C23	−129.6 (2)
C6—N6—C1—O1	−177.26 (19)	C21—N12—C17—N11	−178.55 (16)
C6—N6—C1—N1	2.6 (3)	C21—N12—C17—C18	−0.6 (3)
C1—N6—C6—N5	−176.04 (17)	C17—N12—C21—C20	−0.7 (3)
C1—N6—C6—N7	4.0 (3)	C26—N13—C22—N11	−176.98 (18)
C4—N7—C2—N1	178.92 (16)	C26—N13—C22—C23	0.0 (3)
C4—N7—C2—N2	−1.3 (3)	C22—N13—C26—C25	−0.6 (3)
C6—N7—C2—N1	3.2 (3)	N8—C7—C8—C9	174.24 (17)
C6—N7—C2—N2	−176.95 (17)	N9—C7—C8—C9	0.2 (3)
C2—N7—C4—N3	5.9 (3)	C7—C8—C9—C10	−0.1 (3)
C2—N7—C4—N4	−173.37 (17)	C8—C9—C10—C11	−0.3 (3)
C6—N7—C4—N3	−178.42 (17)	C9—C10—C11—N9	0.6 (3)
C6—N7—C4—N4	2.3 (3)	N8—C12—C13—C14	177.29 (19)
C2—N7—C6—N5	172.85 (17)	N10—C12—C13—C14	0.3 (3)
C2—N7—C6—N6	−7.2 (3)	C12—C13—C14—C15	−0.3 (3)
C4—N7—C6—N5	−2.8 (3)	C13—C14—C15—C16	0.4 (3)
C4—N7—C6—N6	177.17 (17)	C14—C15—C16—N10	−0.5 (3)
C7—N8—C3—N2	10.0 (3)	N11—C17—C18—C19	179.74 (17)
C7—N8—C3—N3	−171.03 (17)	N12—C17—C18—C19	1.9 (3)
C12—N8—C3—N2	178.50 (16)	C17—C18—C19—C20	−1.9 (3)
C12—N8—C3—N3	−2.5 (3)	C18—C19—C20—C21	0.7 (3)
C3—N8—C7—N9	−134.91 (18)	C19—C20—C21—N12	0.7 (3)
C3—N8—C7—C8	50.4 (3)	N11—C22—C23—C24	177.43 (19)
C12—N8—C7—N9	55.8 (2)	N13—C22—C23—C24	0.7 (3)
C12—N8—C7—C8	−118.8 (2)	C22—C23—C24—C25	−0.7 (3)
C3—N8—C12—N10	−119.0 (2)	C23—C24—C25—C26	0.1 (3)
C3—N8—C12—C13	63.7 (3)	C24—C25—C26—N13	0.6 (3)

### Hydrogen-bond geometry (Å, º)

Cg1 and Cg2 are the centroids of the N12/C17–C21 and N13/C22–C26 rings, respectively.

**Table:**

*D*—H···*A*	*D*—H	H···*A*	*D*···*A*	*D*—H···*A*
N1—H1*N*···O3^i^	0.87 (2)	1.84 (2)	2.706 (2)	169 (2)
O3—H1*O*···O2	0.89 (4)	1.92 (3)	2.755 (2)	157 (3)
O3—H2*O*···N12^ii^	0.89 (3)	1.99 (3)	2.859 (2)	165 (3)
O4—H3*O*···N6^iii^	0.94 (4)	2.04 (4)	2.904 (3)	151 (3)
O4—H4*O*···N5^iii^	1.02 (3)	2.55 (4)	3.206 (3)	122 (2)
C9—H9···N10^iv^	0.95	2.55	3.313 (3)	137
C21—H21···O2^v^	0.95	2.41	3.291 (3)	154
C23—H23···O1^vi^	0.95	2.55	3.426 (2)	153
C27—H27*B*···*Cg*2	0.98	2.76	3.647 (3)	150
C28—H28*A*···*Cg*1^vii^	0.98	2.73	3.463 (2)	132

Symmetry codes: (i) −*x*, −*y*+1, −*z*+1; (ii) *x*, *y*+1, *z*; (iii) −*x*+1, −*y*, −*z*+1; (iv) −*x*−1, −*y*+1, −*z*+1; (v) *x*, *y*−1, *z*; (vi) −*x*, −*y*, −*z*+1; (vii) −*x*+1, −*y*, −*z*+2.
